# Right middle and lower bilobectomy using a modified fissureless technique: a case report

**DOI:** 10.1186/s44215-026-00262-5

**Published:** 2026-05-12

**Authors:** Eiji Narusawa, Toshiteru Nagashima, Yoichi Ohtaki, Natsuko Kawatani, Tomohiro Yazawa, Ryohei Yoshikawa, Masazumi Koike, Ken Shirabe, Seshiru Nakazawa

**Affiliations:** 1 Department of General Surgical Science, Gunma University Graduate School of Medicine, Maebashi, Japan; 2 Division of Thoracic Surgery, Gunma Prefectural Cancer Center, Ota, Japan; 3 Department of Thoracic Surgery, National Cancer Center Hospital East, Kashiwa, Japan; 4 Department of Human Pathology, Gunma University Graduate School of Medicine, Maebashi, Japan; 5 Clinical Department of Pathology, Gunma University Hospital, Maebashi, Japan

**Keywords:** Fissureless lobectomy, Bilobectomy, Fissureless technique

## Abstract

**Background:**

Fissureless lobectomy has been shown to prevent air leaks in patients with fused or incomplete interlobar fissures or emphysema. The technique is also beneficial in advanced lung cancers involving adjacent lobes, where interlobar manipulation is technically difficult. Although this technique is widely used in standard lobectomies, reports on bilobectomy remain scarce.

**Case presentation:**

A 57-year-old woman with left-sided tongue cancer (pT2N0M0, Stage II) underwent subtotal glossectomy followed by postoperative concurrent chemoradiotherapy. Eighteen months after the surgery, imaging revealed a mass in the right lower lobe. Bronchoscopy confirmed squamous cell carcinoma, and the patient was referred to our department with suspected pulmonary metastasis.

Over the 3 weeks following initial presentation, the lung tumor enlarged rapidly. The tumor was centered in the lower lobe with invasion of the middle and upper lobes, and the distal lung parenchyma was complicated by obstructive pneumonia. Although antibiotics were administered, the fever and inflammatory markers persisted; thus, we decided to proceed with surgery for infection control. Due to the presence of incomplete lung fissure and the lower lobe tumor invading the other lobes, pneumonectomy was the initial option. However, to preserve lung capacity, we decided to perform a fissure-last right middle and lower bilobectomy with division of the interlobar pulmonary artery prior to resection of the bronchus. The patient was discharged 16 days after surgery without complications.

**Conclusions:**

We report a case of bilobectomy using a modified fissureless technique and describe the technical details of how we dissect the pulmonary artery prior to resection of the bronchus. Being familiar with the fissureless bilobectomy technique is an important option for avoiding right pneumonectomy.

**Supplementary Information:**

The online version contains supplementary material available at 10.1186/s44215-026-00262-5.

## Background

The fissureless technique helps prevent postoperative air leaks after pulmonary lobectomy when the interlobar fissure is incomplete or fused [1, 2]. This technique can also be applied in the context of lung cancers with interlobar invasion or those in incomplete or fused fissures[3]. Two distinct approaches to fissureless lobectomy have been described: the “fissure-last lobectomy,” in which the hilar structures are divided first and the fissure is divided as the final step, and the “fissure-first lobectomy,” in which this sequence is reversed [4, 5]. Despite the widespread use of fissureless lobectomy, only few reports to date have described the detailed technique for bilobectomy using fissureless technique [6–8]: one reported a fissure-first approach and two reported a fissure-last approach. Our case involved fissure-last bilobectomy in which the interlobar pulmonary artery was divided before the bronchus intermedius was addressed. We described the technical details of fissure-last right middle and lower bilobectomy with dissection of the pulmonary artery prior to resection of the bronchus.

## Case presentation

**Fig. 1:**
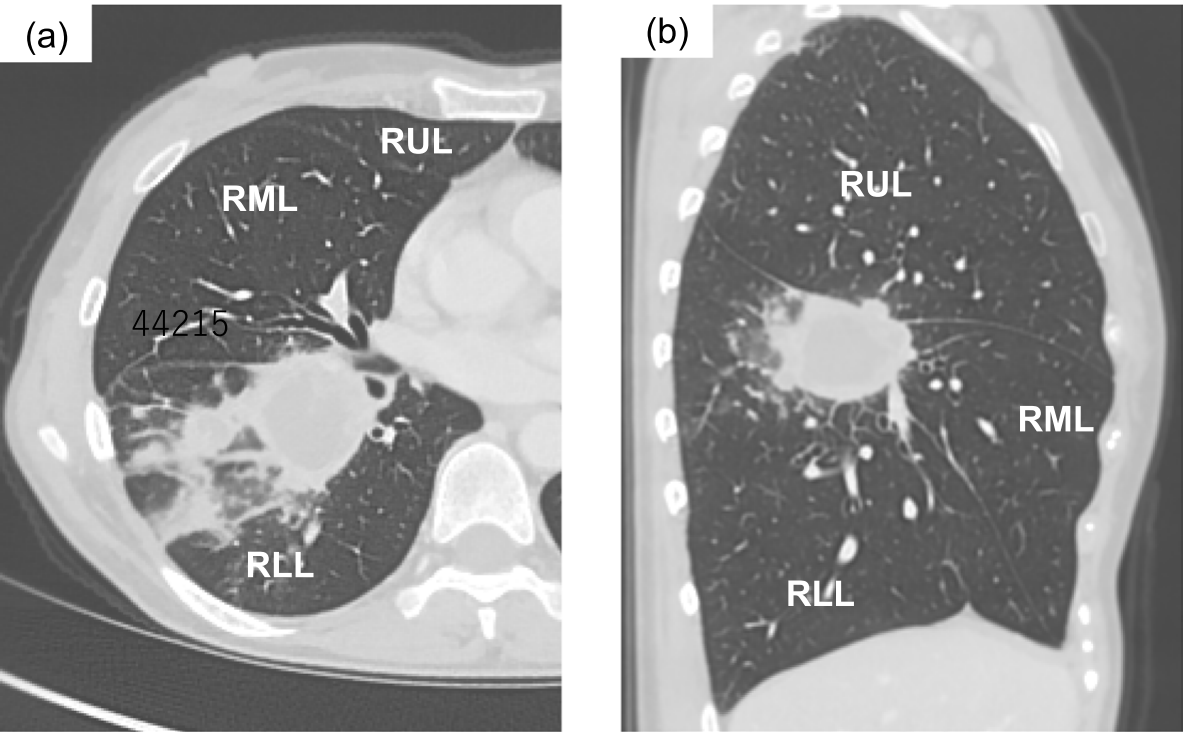
Preoperative chest computed tomography (CT). **a** Axial CT image showing a lung mass predominantly involving the right lower lobe with extension toward the middle lobe. **b** Sagittal reconstruction showing the mass centered in the superior segment (S6) and located at the confluence of the three lobes

**Fig. 2:**
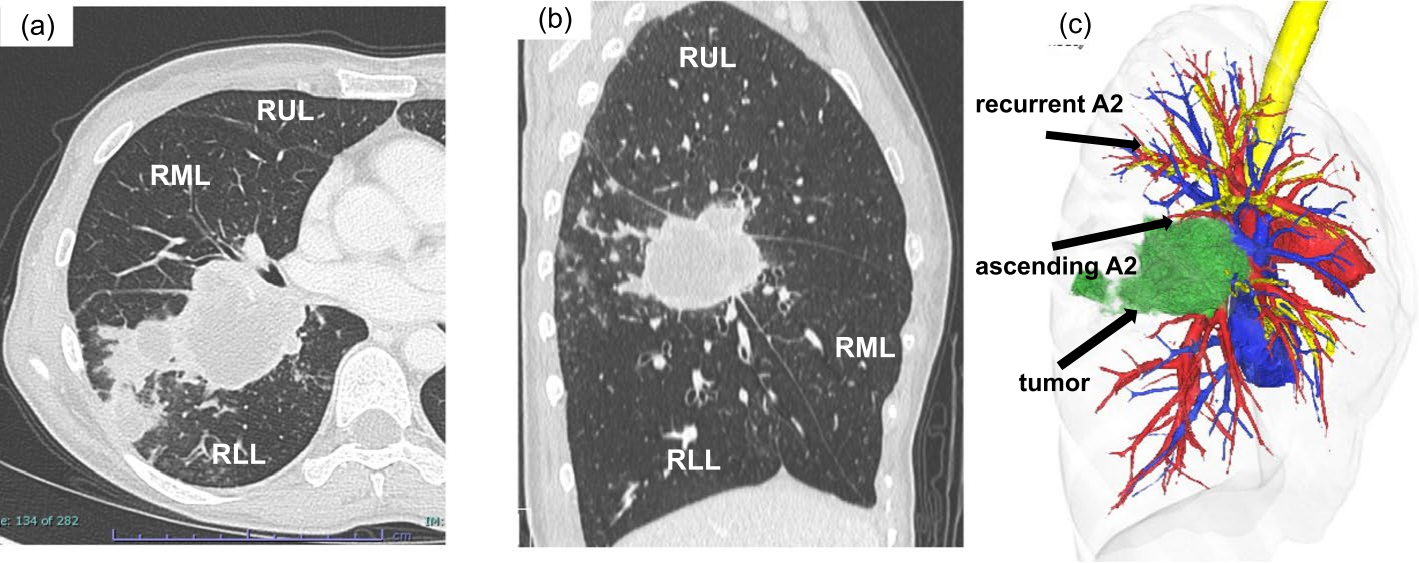
Chest computed tomography (CT) and preoperative three-dimensional CT three weeks after the initial scan (**a**) Follow-up chest CT obtained three weeks after the initial scan demonstrates rapid enlargement of the tumor. **b** Sagittal reconstructions show tumor invasion in the middle and upper lobes. **c** The tumor has invaded to the branching of the ascending pulmonary artery. A recurrent A2 branched from the upper lobe trunk of the pulmonary artery

A 57-year-old woman with a 35 pack-year smoking history presented for follow-up after treatment for tongue cancer. The patient had underwent surgery for tongue cancer, followed by adjuvant chemoradiotherapy, and remained disease-free for 18 months. The tongue cancer was diagnosed as pT2N0M0 (stage II). A follow-up chest computed tomography (CT) revealed a 4.5—cm mass in the right lower lobe that was not present on imaging performed 12 months prior (Fig. 1). Bronchoscopy confirmed a diagnosis of squamous cell carcinoma. Three weeks after the diagnosis, the patient developed fever due to an intratumoral lung abscess, with elevated inflammatory markers (WBC 8.4 × 10^3^/μL, CRP 4.28 mg/dL), and garenoxacin (400 mg/day) was initiated. Over the subsequent three weeks, the tumor enlarged rapidly, invading from the lower lobe to the middle and upper lobes, and the distal lung developed obstructive pneumonia (Fig. 2a, b). Further CT from the chest to the abdomen, FDG-PET, or brain MRI revealed no additional metastatic lesions. Inflammatory markers worsened despite antibiotic therapy (WBC 10.0 × 10^3^/μL, CRP 8.12 mg/dL). Based on the presumed solitary nature of the lesion, its potential for local control, and inadequate response to antibiotic therapy, surgery was considered. The patient was informed of this plan, understood it, and agreed to the proposed treatment. However, the enlarged hilar lymph nodes and tumor invasion extending to the branching of the ascending pulmonary artery indicated that a right middle and lower bilobectomy would be required, with the possibility of right pneumonectomy. Given the rapid tumor progression associated with infection, the case was at risk of evolving into an oncologic emergency. If preservation of the upper lobe was not feasible, pneumonectomy was considered as a realistic and acceptable option.

We generated a 3D-CT reconstruction image which confirmed the presence of a recurrent A2 (Fig. 2c). Because preservation of the ascending A2 was difficult due to tumor invasion (Supplementary Fig. 1), we planned to divide the ascending A2 if middle and lower lobectomy was feasible, as the recurrent A2 would maintain arterial perfusion to the remaining lobe. In addition, no tumor invasion was observed in the proximal interlobar pulmonary artery, and we anticipated that it could be safely secured intraoperatively.

After switching to sulbactam/ampicillin (1200 mg/day) and administering it for 5 days, a posterolateral thoracotomy was performed through the fifth intercostal space. The lower lobe tumor had invaded the upper and middle lobes, rendering an interlobar approach technically unfeasible; therefore, instead of a right pneumonectomy, a bilobectomy was conducted using the modified fissureless technique.

**Fig. 3:**
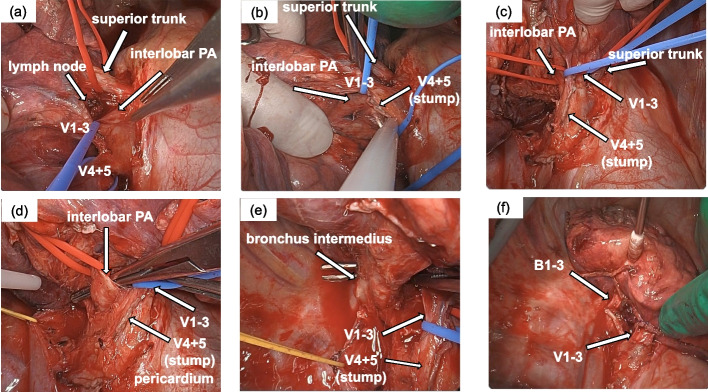
Intraoperative findings and procedure. **a** The lymph node could be safely dissected from the intermediate pulmonary artery. **b** The interlobar pulmonary artery is encircled behind the divided middle lobe vein. **c** The red vessel loop initially encircled the interlobar pulmonary artery from the caudal side of the upper lobe vein. The red vessel loop was then passed behind the upper lobe vein, to the dorsal side. **d** The interlobar pulmonary artery is safely divided from the anterior aspect. **e** The bronchus intermedius is encircled after division of the interlobar pulmonary artery. **f** Operative view after middle and lower bilobectomy

After encircling the inferior pulmonary vein, superior pulmonary vein, and the superior trunk, we divided the middle lobe vein and encircled the interlobar pulmonary artery right before branching of ascending A2 (Fig. 3a,b). The inferior pulmonary vein was then divided, and the lung was retracted cranially. The vessel loop around the pulmonary artery was passed behind the upper lobe vein and pulled dorsally. This maneuver enabled the safe division of the pulmonary artery from the anterior side, allowing transection of the pulmonary artery proximal to the ascending A2 branch (Fig. 3c,d; Additional file 1). As the posterior hilar interlobar lymph node (#11 s region) could be dissected from the intermediate bronchus, the intermediate bronchus was transected (Fig. 3e). Finally, while palpating the tumor, we determined the resection margin and divided the interlobar plane with a stapler, concomitantly resecting a part of the S2 segment and completing bilobectomy (Fig. 3f; Additional file 2). The bronchial stump was covered with a pedicled pericardial fat pad. The operative time was 198 min, and the blood loss was 168 mL. Antibiotic therapy was continued for 5 days postoperatively, during which inflammatory markers showed improvement (WBC 7.1 × 10^3^/μL, CRP 4.51 mg/dL). The patient was discharged on postoperative day 16 without any complications. The postoperative pathological diagnosis was squamous cell carcinoma, with lymph node metastases showing conglomeration due to direct tumor invasion. Vascular, perineural, and bronchial invasion were identified; however, both the bronchial and surgical margins were negative, indicating complete resection. At the latest follow-up, 18 months after surgery, multiple metastases from tongue cancer-including bone, brain, and pulmonary metastases-were identified, and the patient was receiving treatment with immune checkpoint inhibitors.

## Discussion

Fissureless technique is useful for preventing air leakage [1, 2]. This technique may also be applicable to advanced lung cancers extending across adjacent lobes, in which conventional interlobar dissection is not feasible [3]. Although fissureless technique is widely used, bilobectomy using fissureless technique is rarely reported [6–8]. Additionally, to the best of our knowledge, no previous reports have described anterior management of the interlobar pulmonary artery by modifying the use of a vessel loop to enable division of the artery prior to addressing the bronchus intermedius.

Initially, given the rapid progression of the tumor and the concern that station #11 s interlobar lymph nodes could not be dissected away from the bronchus intermedius, we considered that right pneumonectomy might be necessary. However, by using the fissure-last approach, we preserved the right upper lobe.

In this case, the infected tumor was located adjacent to the fused interlobar fissures, and conventional fissure dissection may have caused tumor seeding or bacterial spillage. Therefore, we adopted a fissureless technique. The main drawback of fissureless lobectomy is the pulmonary artery injury [5]. In our patient, the interlobar pulmonary artery was safely divided from the anterior side under adequate visualization from multiple directions, including anterior and cranial views, before dissecting the bronchus, as described above.. This arterial control subsequently allowed a safe management of the bronchus intermedius. In this case, the interlobar pulmonary artery could be safely controlled. However, when such control is difficult, proximal control of the main pulmonary artery should be considered. Moreover, the presence of recurrent A2 branch allowed us to sacrifice the ascending A2; therefore, we were able to control and divide the interlobar pulmonary artery trunk proximal to the ascending arterial branches. If the absence of a recurrent A2 pulmonary arterial branching pattern, preservation of segment S2 might not have been feasible.

In this case, the bulky and enlarged station #11 s lymph node made it difficult to securely mobilize and obtain an adequate length of the bronchus intermedius. To avoid right pneumonectomy, a relatively radical dissection around the bronchus is unavoidable. However, such a manipulation would carry an unacceptably high risk of pulmonary artery injury if the interlobar pulmonary artery was not dissected first. Therefore, we deliberately divided the interlobar pulmonary artery first, to create a wider and safer working space for radical dissection of lymph node #11 s from the bronchus. Moreover, even if the bronchus intermedius had been transected first, adequate exposure of the hilar structures would not have been achieved due to the large tumor volume [6], and this maneuver could have increased tension on the pulmonary artery. Furthermore, by applying a fissure-last technique for the final division of the interlobar plane, we could confirm an adequate surgical margin while carefully avoiding inadvertent stapling of the preserved upper lobe vein.

Pneumonectomy carries a high risk of serious complications, with a 30-day mortality of approximately 4.2% and 90-day mortality of 6.9% [9]. The health-related quality of life after pneumonectomy has been reported to be significantly lower than that in the general population. In particular, worsening dyspnea has been noted in women undergoing right-sided pneumonectomy [10]. Preservation of the pulmonary reserve facilitates the administration of postoperative chemotherapy and radiotherapy.

In this case, the patient’s clinical course led us to suspect that the pulmonary lesion was a metastasis of tongue cancer. Tongue cancer with pulmonary metastasis has poor prognosis and generally requires multimodal therapy [11]. Even if the lung lesion can be controlled, metastases to other sites may occur. Thus, it is essential to preserve the patient’s capacity to tolerate subsequent treatments. Currently, the patient has distant metastases from the tongue cancer, including bone metastases, brain metastases, and multiple pulmonary metastases, and is undergoing immune checkpoint inhibitor therapy. We believe that preserving the right upper lobe by performing a fissureless bilobectomy helps maintain treatment tolerability.

## Conclusion

In cases in which the cancer crosses the interlobar fissure or in which enlarged interlobar lymph nodes would otherwise necessitate pneumonectomy, bilobectomy using the modified fissureless technique described here may offer a means of avoiding pneumonectomy.

## Supplementary Information

Supplementary Material 1.

Supplementary Material 2.

Supplementary Material 3.

## Data Availability

The dataset supporting the conclusion of this article is included within the article and its additional file.

## Abbreviation

CT: Computed tomography
