# Supplementary material for: Right middle and lower bilobectomy using a modified fissureless technique: a case report
Source: Gen Thorac Cardiovasc Surg Cases. 2026 May 12;5:26. doi: 10.1186/s44215-026-00262-5 (PMC13352725; doi:10.1186/s44215-026-00262-5)
Supplement: Supplementary file 3 — Supplementary Material 3. [file 44215_2026_262_MOESM3_ESM.docx]

Supplementary Figure 1.


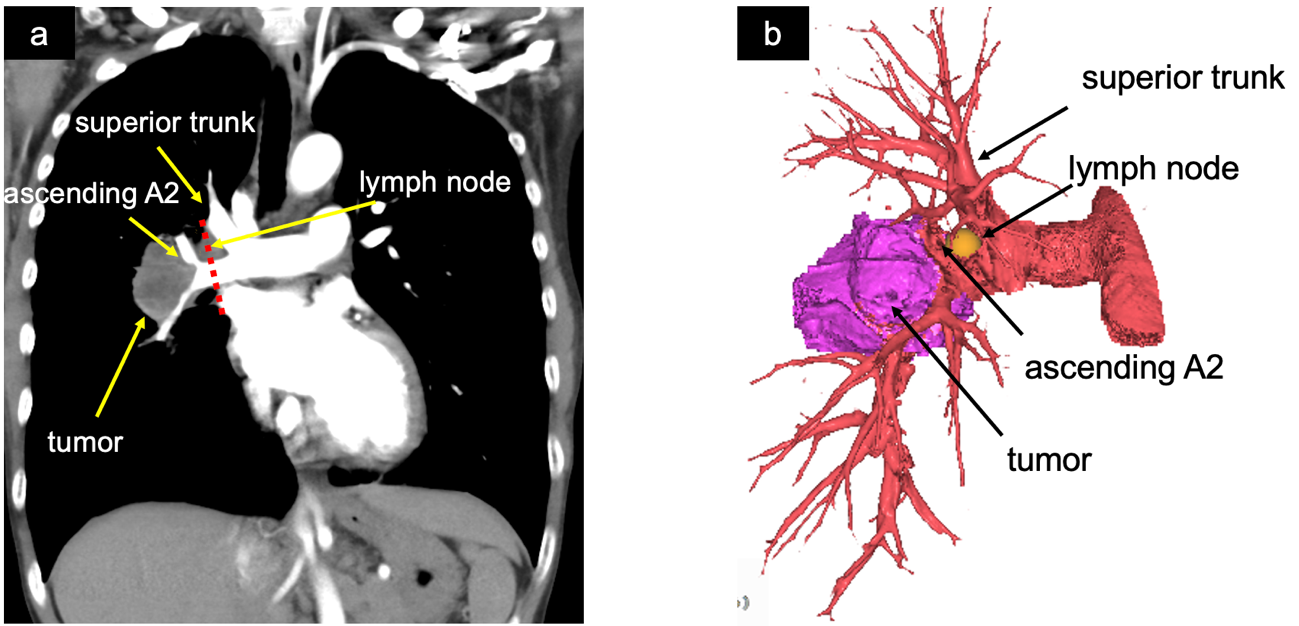


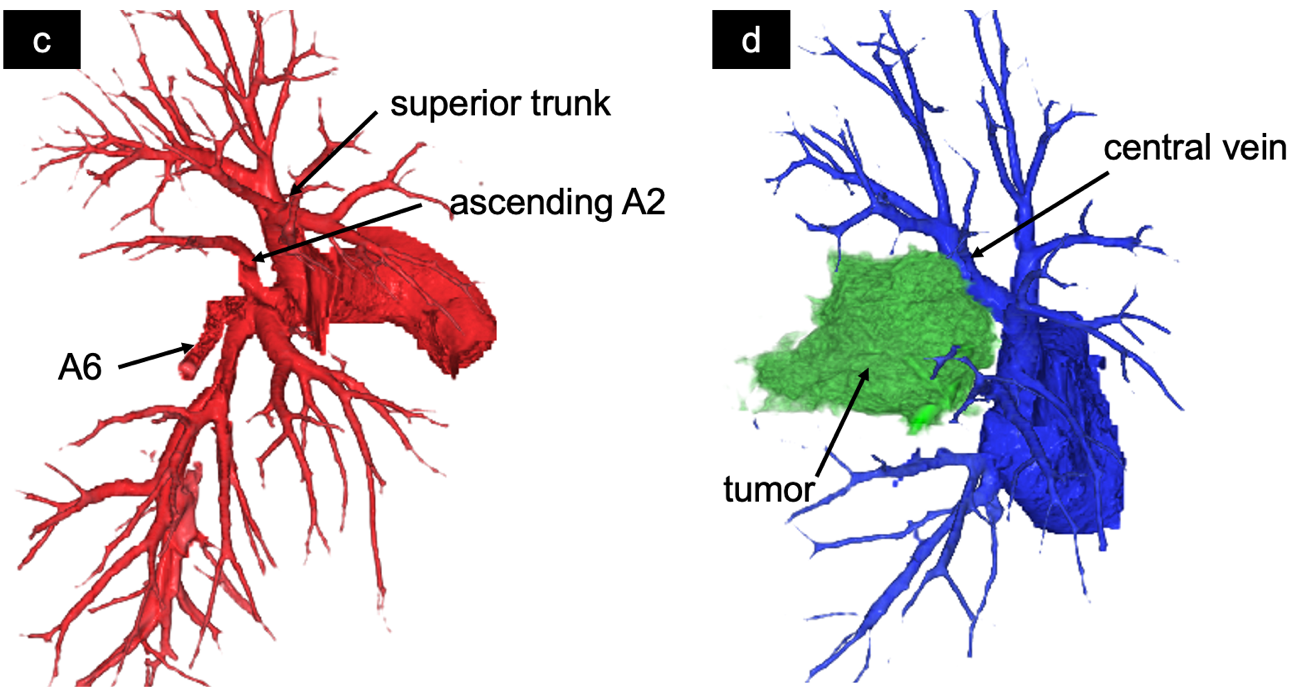


(a) Coronal computed tomography (CT) image. The red dashed line indicates the planned division line of the pulmonary artery in this case.
(b) Three-dimensional CT image showing the spatial relationship among the pulmonary artery, tumor, and enlarged lymph nodes.

(c) Three-dimensional CT image of the pulmonary artery only, showing that A2b branches near A6.

(d) Three-dimensional CT image showing the relationship between the pulmonary vein and tumor. The tumor is in close proximity to the central vein.
